# Recombinant rat heat shock protein 70 (rrHSP70) may promote remyelination by regulating microglia/macrophages and oligodendrocytes in a toxin-induced demyelination animal model

**DOI:** 10.3389/fimmu.2026.1750555

**Published:** 2026-07-23

**Authors:** Jian Song, Jiayan Wang, Yuanzhi Li, Hui Wang, Mei Li, Fei Huang, Xiaoming Zhang, Ding Wan, Jiahao Ye, Keyu Zhou, Lu Yang, Haining Li, Chun Zhang, Tao Sun, Qiaofeng Wan, Shaozhang Hou, Changsen Leng, Peng Wang

**Affiliations:** 1 Ningxia Key Laboratory of Cerebrocranial Diseases, School of Basic Medical Sciences, Ningxia Medical University, Yinchuan, Ningxia, China; 2 College of Traditional Chinese Medicine, Ningxia Key Laboratory of Cerebrocranial Diseases, Ningxia Medical University, Yinchuan, Ningxia, China; 3 Emergency Department of Ningxia Medical University General Hospital; Key Laboratory of Craniocerebral Diseases, Ningxia Medical University, Yinchuan, Ningxia, China; 4 Center for Translational Neurodegeneration and Regenerative Therapy, Shanghai Tongji Hospital Affiliated to Tongji University School of Medicine, Shanghai, China; 5 Department of Neurosurgery, General Hospital of Ningxia Medical University; Ningxia Key Laboratory of Cerebrocranial Diseases, Ningxia Medical University;, Yinchuan, Ningxia, China; 6 School of Clinical Medicine, Ningxia Medical University, Yinchuan, Ningxia, China; 7 Department of Neurology, General Hospital of Ningxia Medical University, Ningxia Key Laboratory of Cerebrocranial Diseases, Ningxia Medical University;, Yinchuan, Ningxia, China; 8 State Key Laboratory of Oncology in South China, Guangdong Provincial Clinical Research Center for Cancer, Department of Thoracic Surgery, Guangdong Esophageal Cancer Institute, Sun Yat-sen University Cancer Center, Guangzhou, China; 9 Ningxia Key Laboratory of Cerebrocranial Diseases, Key Laboratory of Dryness Syndrome in Chinese Medicine, Ministry of Education, School of Basic MedicalSciences,Ningxia Medical University, Yinchuan, Ningxia, China

**Keywords:** multiple sclerosis (MS), oligodendrocytes (OLGs), phagocytosis of microglia and macrophages, recombinant rat heat shock protein 70 (rrHSP70), remyelination

## Abstract

Heat shock protein 70 (HSP70) can assemble with other extracellular matrix proteins to form deoxycholate-insoluble aggregates in multiple sclerosis (MS) lesions, impairing oligodendrocyte (OLG) maturation and remyelination. However, soluble HSP70 supports myelination. HSP70 may also promote bone marrow-derived macrophages (BMDMs) toward classically and alternatively activated phenotypes, which may regulate OLG maturation. Given the roles of HSP70 in OLG maturation and in shaping microglial/BMDM phenotypes in MS lesions, we investigated how HSP70 in microglia/macrophages regulates OLG maturation in a toxin-induced demyelination model. Our results suggest that rrHSP70 may maintain HSP70 expression in microglia and BMDMs *in vitro*. Tandem mass tag (TMT)-labeled quantitative proteomic, post-translational modification, and phagocytosis analyses showed that rrHSP70 may induce Fc gamma R-mediated phagocytosis in microglia and BMDMs, enabling rrHSP70 engulfment and enhancing heat shock factor 1 (HSF-1) phosphorylation. Blocking or inhibiting CD45 activity reduced rrHSP70 phagocytosis and HSF-1 phosphorylation in microglia. These findings suggest that rrHSP70 may induce Fc gamma R-mediated phagocytosis in microglia and BMDMs, leading these cells to phagocytose rrHSP70 and enhance HSF-1 phosphorylation, thereby maintaining HSP70 expression *in vitro*. Furthermore, HSP70 levels increased during early demyelination in the animal model, suggesting that HSP70 may induce microglial/macrophage phagocytosis to remove factors that inhibit remyelination, thereby facilitating later remyelination. rrHSP70 likely promoted OLG maturation both *in vivo* and *in vitro*, as well as remyelination, possibly by enhancing myelin basic protein (MBP) expression and facilitating phosphorylated MBP redistribution. Overall, context-dependent HSP70 upregulation may be crucial for enhancing remyelination in MS lesions.

## 1 Introduction

Multiple sclerosis (MS) is a chronic immune-mediated demyelinating disease of the central nervous system (CNS) characterized by focal demyelination, chronic inflammation, gliosis, and oligodendrocyte (OLG) loss (1). In the CNS, OLG precursor cells (OPCs) differentiate into mature OLGs, which support myelination and remyelination (2). Remyelination maintains efficient saltatory conduction and provides essential metabolic support for neurons (3–5). In MS, OPCs migrate to lesion sites and differentiate into OLGs to promote remyelination early in disease; however, this repair process fails as MS progresses (3). Moreover, inflammatory responses and dysregulated extracellular matrix (ECM) molecules, such as fibronectin aggregates (aFn), may impair OPC differentiation and maturation, leading to remyelination failure in MS (6, 7). Therefore, enhancing remyelination requires removing intrinsic and extrinsic factors that inhibit OLG maturation during MS progression.

In MS, fibronectin modulates heat shock protein 70 (HSP70) and other ECM proteins into deoxycholate-insoluble aFn, impairing OLG maturation and remyelination (8, 9). Furthermore, HSP70 forms deoxycholate-insoluble aggregates in the ECM of MS lesions, which may shift microglia and macrophages toward a phenotype associated with impaired remyelination (8). In MS and experimental autoimmune encephalomyelitis (EAE), an animal model of MS, immune responses induce HSP70 overexpression within lesions (10–12). HSP70 is a stress-induced chaperone protein that functions intracellularly to support cytoprotection and extracellularly to modulate immune responses in MS (13–15). One study suggested that HSP70 gene polymorphisms are associated with MS development (16). Some studies have also shown that HSP70 overexpression in MS protects CNS cells against inflammatory injury (12, 17, 18). However, some evidence suggests that overexpression of the inducible form of HSP70, driven by an inflammatory microenvironment, enhances the presentation of myelin basic protein (MBP), a potential autoantigen, contributing to MS development (19). Therefore, high HSP70 expression may contribute to MS pathogenesis through inflammatory signaling and/or myelin autoantigen presentation after tissue injury and cell death, supporting a context-dependent role of HSP70 in MS.

However, intracellular HSP70 upregulation reduces disease incidence and neuronal damage and improves clinical signs in the EAE animal model (20). Notably, neurons poorly induce HSP70 expression; therefore, HSP70 may be transferred from glial cells, including microglia (11). Previous studies have shown that HSP70 is primarily produced by reactive astrocytes, OLGs, and microglia in the CNS (21, 22). Glial-cell stimulation with pro-inflammatory cytokines induces HSP70 expression, mainly in OLGs (23). Therefore, neuronal uptake of glia-derived HSP70 may provide compensatory neuroprotection. Inducible HSP70 expression promotes proteasomal degradation and prevents neuregulin-induced demyelination (24). Moreover, a previous study showed that recombinant human heat shock protein 70 (rhHSP70) promoted myelination and OLG maturation in the cerebellum of a genetically modified animal model (25). Additionally, HSP70 facilitates nuclear import carrier activity, improving OLG differentiation and maturation and CNS myelination (26), which contrasts with its aggregation with other ECM proteins and inhibition of remyelination in MS. Therefore, clarifying the role of HSP70 as an insoluble ECM-associated protein in remyelination is important in MS.

Considering elevated HSP70 levels in MS lesions, this study investigated the role of HSP70 in OLG maturation and in shaping microglia/BMDM phenotypes that influence OLG maturation in MS lesions. We hypothesized that HSP70 may regulate microglia/macrophages and OLGs during remyelination in MS lesions. In this study, administration of recombinant rat heat shock protein 70 (rrHSP70) at the onset of remyelination in a toxin-induced demyelination animal model likely promoted OLG maturation compared with controls. Additionally, rrHSP70 may promote OLG maturation by enhancing MBP expression and facilitating phosphorylated MBP (pMBP) redistribution. Furthermore, rrHSP70 may induce Fc gamma R-mediated phagocytosis in microglia and BMDMs, enabling rrHSP70 engulfment, enhancing heat shock factor 1 (HSF-1) phosphorylation, and maintaining HSP70 expression *in vitro*. The toxin-induced demyelination animal model showed increased HSP70 levels during early demyelination, with similar levels in the control and remyelination phases. These findings suggest that rrHSP70 may stimulate its own phagocytic uptake to regulate HSF-1 phosphorylation in microglia/macrophages, thereby enhancing HSP70 expression and possibly promoting OLG maturation during remyelination in a toxin-induced demyelination animal model.

## 2 Materials and methods

### 2.1 Primary cell cultures

#### 2.1.1 Microglia

Primary microglial cultures were prepared using a previously established method with minor modifications (27). Briefly, single-cell suspensions were obtained from the forebrains of 1–2-day-old Sprague–Dawley (SD) rats by mechanical and enzymatic (papain) digestion. After culture for 10–12 days in poly-L-lysine (PLL, 5 mg/mL, Solarbio, Beijing, China)-coated tissue culture flasks (Servicebio, Wuhan, China), microglia and OPCs were detached by rotation on an orbital shaker (Zhichu Company, Shanghai, China). Microglia were detached by shaking at 150 rpm for 2 h and cultured in microglial culture medium [Dulbecco’s Modified Eagle Medium (DMEM) (Gibco, Grand Island, USA); 10% fetal bovine serum (Sartorius, Gottingen, Germany); 1% penicillin-streptomycin (Gibco, Grand Island, USA); 1% glutamine (Gibco, Grand Island, USA); and rat recombinant macrophage colony-stimulating factor (M-CSF, 10 ng/mL, Peprotech, Cranbury, USA)] at a density of 2.0×10^6^ cells per 10 cm dish. After 3 days of culture, microglia were plated in 6-well plates at a density of 1.0×10^6^ cells per well (in 1 mL). Microglia were left untreated or treated with rrHSP70 (500 ng/mL, Enzo Biochem) in conditioned microglial medium (CMM) [DMEM (Gibco, Grand Island, USA); 1% penicillin-streptomycin (Gibco, Grand Island, USA); and 1% glutamine (Gibco, Grand Island, USA)] for 48 h [TMT-labeled quantitative proteomic analysis, parallel reaction monitoring (PRM) analyses, and immunocytochemistry] or 30 min (post-translational modification [PTDM] analysis).

For OPC culturing and western blotting of HSP70 expression in culture medium, microglia were incubated with rrHSP70 (500 ng/mL, Enzo Biochem) or aFn (100 μg/mL) in CMM for 24 h. The medium was then replaced, and microglia were cultured for another 24 h to remove residual rrHSP70 and aFn. Finally, CMM was collected for OPC culture and western blotting of HSP70 expression in culture medium.

#### 2.1.2 OPCs

The remaining flasks were shaken overnight at 150 rpm on an orbital shaker to isolate OPCs. For maturation assays, cells were plated at a density of 0.8×10^5^ cells per well on 24-well plates with glass (NEST Biotech, Wuxi, China). Isolated OPCs were cultured for 2 days in OPC culture medium containing 96% Neurobasal (Gibco, Grand Island, USA), 2% B27 (Gibco), 1% glutamine (Gibco), 1% penicillin-streptomycin (Gibco), and 0.02% T3 (Gibco), supplemented with growth factors [FGF-2 (10 ng/mL, Peprotech) and PDGF-AA (10 ng/mL, Peprotech)]. To obtain mature OLGs, maturation was initiated by withdrawing the growth factors. OLGs were then left untreated, treated with rrHSP70 (500 ng/mL, Enzo Biochem) in culture medium for 3 or 7 days, or exposed to microglia/macrophage-conditioned medium collected over 72 h for maturation assays.

### 2.2 Bone marrow-derived macrophages

Bone marrow-derived macrophages were isolated from 1–2-day-old SD rats as previously described, with minor modifications. Bone marrow was flushed with sterile phosphate-buffered saline (PBS). Suspensions were centrifuged at 1000 rpm for 5 min, and cell pellets were resuspended in BMDM culture medium [RPMI medium (Gibco), 10% fetal bovine serum (Sartorius), 1% sodium pyruvate (Gibco), 1% penicillin-streptomycin (Gibco), and M-CSF (10 ng/mL, Peprotech)] and seeded in 10 cm dishes (Servicebio) at a density of 2.0×10^6^ cells per dish for 7 days. Macrophages were then plated in 6-well plates at a density of 1.0×10^6^ cells per well and left untreated or treated with rrHSP70 (500 ng/mL, Enzo Biochem) in macrophage-conditioned medium [RPMI medium (Gibco), 10% fetal bovine serum (Sartorius), 1% sodium pyruvate (Gibco), and 1% penicillin-streptomycin (Gibco)] for 30 min (PTDM analysis) or 48 h (TMT-labeled quantitative proteomic analysis and PRM analyses).

For OPC culture and western blotting of HSP70 expression in culture medium, macrophages were incubated with rrHSP70 (500 ng/mL, Enzo Biochem) or aFn (100 μg/mL) in macrophage-conditioned medium for 24 h. The medium was then replaced, and macrophages were cultured for another 24 h in macrophage-conditioned medium to remove residual rrHSP70 and aFn. Macrophage-conditioned medium was then collected for OPC culture and western blotting of HSP70 expression in culture medium.

### 2.3 aFn generation

Astrocytes from primary cell cultures were stimulated with poly(I:C) (50 μg/mL, GE Healthcare, Chicago, USA) and lipopolysaccharide (50 μg/mL, Sigma–Aldrich) to generate aFn as previously described, with minor modifications (28). aFn was extracted using DOC buffer [2% deoxycholate, 2 mM EDTA, 5 mM Tris-HCl, pH 7.4, containing a protease inhibitor cocktail (Complete Mini, Sigma–Aldrich)].

### 2.4 TMT-labeled quantitative proteomic analysis

Cells were subjected to TMT-labeled quantitative proteomic analysis by PTM Biolabs Inc. (Hangzhou, Zhejiang, China). Cells obtained from at least three or four independent experiments were analyzed by quantitative proteomics. Samples were lysed in buffer (8 M urea, 2 mM EDTA, and 1% protease inhibitor cocktail) using a high-intensity ultrasonic processor (Scientz Biotechnology, Ningbo, China). After protein concentration was determined using a BCA kit, proteins were digested with trypsin. Briefly, the protein solution was reduced with dithiothreitol (5 mM) for 30 min at 56 °C and alkylated with iodoacetamide (11 mM) for 15 min at room temperature in darkness. Trypsin was then added at a 1:50 trypsin-to-protein mass ratio for overnight digestion, followed by a second digestion at a 1:100 ratio for 4 h. After trypsin digestion, peptides were desalted and dried using a Strata X C18 SPE column (Phenomenex) under vacuum. Peptides were then reconstituted in TEAB (0.5 M) and labeled using a TMT kit/iTRAQ kit, according to the manufacturer’s protocol. Finally, the tryptic peptides were subjected to liquid chromatography–tandem mass spectrometry (LC-MS/MS) analysis.

### 2.5 PRM analysis

PRM analysis was performed by PTM Biolabs Inc. (Hangzhou, Zhejiang, China). Briefly, after protein extraction and trypsin digestion, tryptic peptides were subjected to nanospray ionization followed by MS/MS. Intact peptides were detected in the Orbitrap at a resolution of 35,000. Peptides were selected using an NCE setting of 27, and fragment ions were analyzed in the Orbitrap at a resolution of 17,500. The automatic gain control was set to 3E6, and the maximum IT was set to 20 ms. The isolation window for MS/MS was set to 2.0 m/z. Data were processed using Skyline (v.3.6).

### 2.6 Western blotting

The corpus callosum was dissected from toxin-induced demyelination animal models and washed with PBS. Protein samples were extracted from the corpus callosum by sonication in lysis buffer (50 mM Tris-HCl, 150 mM NaCl, 5 mM EDTA, and pH 7.5) for six 10 s pulses. Protein concentration was measured using a BCA protein assay kit (KeyGEN Biotech, Nanning, China), with bovine serum albumin (BSA) as the standard. Equal amounts of protein (100 μg for brain homogenates) or equal volumes of medium (40 μL) were mixed with SDS-reducing loading buffer, denatured at 95 °C for 5 min, and subjected to western blotting. Primary antibodies are listed in **Table 1**. Signals were detected using the Odyssey Infrared Imaging System (LI-COR, Lincoln, USA) and analyzed using Scion Image software.

**Table 1:** Primary antibodies used during immunohistochemistry, immunocytochemistry and western blot.

Antibody	Manufacture	Dilution western blot	Dilution IHC/ICC
Anti-actin(mAb)	Sigma	1:1000	n.a.
Anti-CNPase(mAb)	Sigma	1:1000	n.a
Anti-HSP70(mAb)	Enzo life science	1:500	1:200
Anti-IBa1(mAb)	Mmillipore	n.a.	1:500
Anti-pMBP(mAb)	Mmillipore	1:500	1:200
Anti-MBP(mAb)	Bio-Red	1:200	1:50
Anti-APC(CC1,mAb)	Calbiochem	n.a.	1:200
Anti-Olig2(mAb)	Mmillipore	n.a.	1:500
Anti-pHSF-1(mAb)	ThermoFisher	n.a.	1:500
Anti-pCD 45(mAb)	ThermoFisher	n.a.	1:200
Anti-CD 45(mAb)	abcam	n.a.	1:500

n.a., not applicable; mAb, monoclonal antibody; pAb, polyclonal antibody; lHC/IC, immunohistochemistry/immunocytochemistry.

### 2.7 PTDM analysis

PTDMs of cells were analyzed by PTM Biolabs Inc. (Hangzhou, Zhejiang, China). Briefly, samples were sonicated three times on ice using a high-intensity ultrasonic processor (Scientz) in lysis buffer (8 M urea, 1% protease inhibitor cocktail). Supernatants were collected, and protein concentration was determined using a BCA kit according to the manufacturer’s instructions. Protein was digested with trypsin as described for TMT-labeled quantitative proteomic analysis. After trypsin digestion, peptides were dissolved in TEAB (0.5 M) and labeled with the respective TMT reagent (based on manufacturer’s protocol, Thermo Fisher Scientific). Samples were then quenched by adding 5% hydroxylamine, desalted with a Strata X C18 SPE column (Phenomenex), and dried by vacuum centrifugation. Peptide mixtures were incubated with IMAC microsphere suspension with vibration in loading buffer (50% acetonitrile/0.5% acetic acid), and elution buffer (containing 10% NH4OH) was added to enrich phosphopeptides. The phosphopeptide-containing supernatant was collected and lyophilized for LC-MS/MS analysis. Gene Ontology (GO) annotation of the proteome was obtained from the UniProt-GOA database (http://www.ebi.ac.uk/GOA/). Kyoto Encyclopedia of Genes and Genomes (KEGG) analysis was conducted using ortholog-cluster information from the KEGG orthology database. KEGG pathways are mainly associated with metabolism, genetic information processing, environmental information processing, cellular processes, rat diseases, and drug development. The KEGG database was used to annotate protein pathways.

### 2.8 Labeling of rrHSP70

rrHSP70 was purchased from Enzo Biochem [details at https://www.enzo.com/product/hsp70-hsp72-rat-recombinant/, Recombinant rat Hsp70/Hsp72, ~72 kDa, UniProt ID P0DMW0 (*Hspa1a*), and P0DMW1 (*Hspa1b)*, produced in *E. coli*] and labeled with the microscale protein labeling kit according to the manufacturer’s protocol (A30006, Thermo Fisher, Waltham, Massachusetts, USA), with minor modifications. rrHSP70 (0.25 μg) was incubated with the reactive dye solution in 1 M sodium bicarbonate under rotation at 100 rpm at room temperature. The conjugation reaction mixture was applied to the center of the resin and centrifuged at 16,000 × g for 1 min to obtain Alexa 488-labeled rrHSP70. Labeled rrHSP70 and Alexa 488-conjugated goat anti-rat IgG were used in the phagocytosis assay.

### 2.9 Phagocytosis assay

Microglia and macrophages were seeded at 1.5×10^5^ cells per well on coverslips in 24-well plates. Cells were incubated with or without rrHSP70 (500 ng/mL, Enzo Biochem) in culture medium for 30 min. After medium replacement, cells were incubated with Alexa 488-labeled rrHSP70 or Alexa 488-conjugated goat anti-rat IgG for 1 h. Cells were then washed three times with PBS for 10 min each. Finally, cells were fixed with 4% paraformaldehyde and subjected to immunocytochemistry.

### 2.10 Immunocytochemistry

Cells were fixed with 4% paraformaldehyde in PBS for 20 min and permeabilized in ice-cold methanol for 5 min. They were then blocked with 1% BSA for 30 min, incubated with primary antibodies (**Table 1**) overnight at 4 °C, and treated with an appropriate Alexa-conjugated secondary antibody overnight at 4 °C. Finally, cells were mounted with 4’,6-diamidino-2-phenylindole (DAPI)-containing mounting medium and examined using a high-resolution digital slide scanning imaging system (Leica). OLGs were identified by morphology, and at least 250 cells were manually scored for MBP positivity. Mature OLGs were defined as MBP-positive cells forming myelin membrane-like structures.

### 2.11 Blocking the CD45 receptor and inhibiting CD45 activation

Microglia were seeded at 1.5×10^5^ cells per well on coverslips in 24-well plates. Cells were incubated with the CD45 primary antibody (1.0 mg/mL, Abcam, Cambridge, UK) or Imatinib (1 μM, sc-267106, Santa Cruz Biotechnology, USA) for 4 h at 37 °C in serum-free culture medium. Cells were then incubated with rrHSP70 (500 ng/mL) or left untreated for 30 min. The medium was then replaced with fresh medium, and cells were incubated with dye-labeled rrHSP70 for 1 h. Finally, cells were fixed with 4% paraformaldehyde and subjected to immunocytochemistry.

### 2.12 Cuprizone-induced demyelination

The cuprizone-induced demyelination model is used primarily to study primary demyelination, subsequent remyelination, and the role of microglia/macrophages in demyelinating lesions without T-cell infiltration. Nine-week-old female C57BL/6 mice were fed standard chow containing 0.2% cuprizone (Research Diets, New Brunswick, USA) for 5 weeks to induce robust, reproducible demyelination in the corpus callosum. After cuprizone withdrawal, mice were fed standard chow for 2 weeks to allow corpus callosum remyelination. Mice were then decapitated under deep anesthesia with 5% isoflurane at specified time points for biochemical and immunohistochemical analyses. The Animal Ethics Committee of Ningxia Medical University (China) approved all experimental procedures.

### 2.13 Stereotactic injections

After 9-week-old female C57BL/6 mice were fed 0.2% cuprizone for 5 weeks, the same surgical procedure was used for saline or rrHSP70 injection (0.5 μg/μL, 3 μL, Enzo Biochem, New York, USA). The following injection coordinates were used: 1 mm left of the bregma, 1 mm anterior to the bregma, and 2.2 mm below the meninges (ML: 1.0, AP: 1.0, and DV: -2.2). Animals were anesthetized with inhaled isoflurane (1–2%) while breathing spontaneously. Mice were then decapitated under deep anesthesia with 5% isoflurane at specified time points for biochemical and immunohistochemical analyses. The Animal Ethics Committee of Ningxia Medical University (China) approved all experimental procedures.

### 2.14 Sudan black B staining

Brain sections were stained with Sudan Black B solution (2.5 mg/mL; Sigma–Aldrich, USA) for 5 min at 37°C. The sections were then washed three times and rinsed with distilled water to stop staining. Finally, sections were imaged using an optical microscope (Leica, Wetzlar, Germany).

### 2.15 Immunohistochemistry

Paraffin-embedded sections were heated at 65 °C for 1 h and deparaffinized twice. Antigen retrieval was performed using Tris-EDTA-containing antigen retrieval solution (Servicebio). Sections were permeabilized with 1% Triton-X-100 at 37 °C for 20 min. They were then blocked with HBSS containing 1 mM HEPES (Gibco), 1% horse serum (Gibco), 10% goat serum (Jackson ImmunoResearch, Lancaster, USA), 0.1% BSA (Servicebio), and 0.25% Triton X-100 (Biotopped, Beijing, China) for 1 h at 37 °C. Sections were incubated with primary antibodies (**Table 1**) overnight at 4 °C, followed by a 2-h incubation with appropriate Alexa-conjugated secondary antibodies. Nuclei were counterstained with DAPI (1 mg/mL, SouthernBiotech, UAB, USA). Sections were analyzed using a confocal laser scanning microscope (Carl Zeiss, Oberkochen, Germany) and a high-resolution digital slide scanning imaging system (Leica, Wetzlar, Germany).

### 2.16 Transmission electron microscopy analysis

Brains were perfused with 2% glutaraldehyde, and lesions were cut into 1 × 1 × 3 mm sections. After fixation in 2% glutaraldehyde for 2 h, lesions were processed using a standard epoxy resin embedding protocol. Ultrathin sections from the lesion center were prepared at the Electron Microscopy Center of Ningxia Medical University and examined with an H-7650 transmission electron microscope. Remyelination in lesions was assessed using the g-ratio (axon diameter divided by the diameter of axons with surrounding myelin sheaths).

### 2.17 Statistics

Statistical analyses were performed using GraphPad Prism 9.0.1 or IBM SPSS Statistics for Windows, version 27 (IBM Corp., Armonk, N.Y., USA). Data are presented as mean ± standard error of the mean. Individual statistical tests and the numbers of independent experiments or animals (n) are provided in the Results section and figure legends. Statistical significance was set at p < 0.05.

## 3 Results

### 3.1 rrHSP70 induces HSP70 expression and secretion in microglia/BMDMs

To identify rrHSP70-regulated molecules in microglia/macrophages, microglia/BMDMs were incubated with or without rrHSP70 or aFn for 48 h. Cells were then collected for TMT-labeled quantitative proteomic analysis, followed by PRM validation.

**Figure 1:**
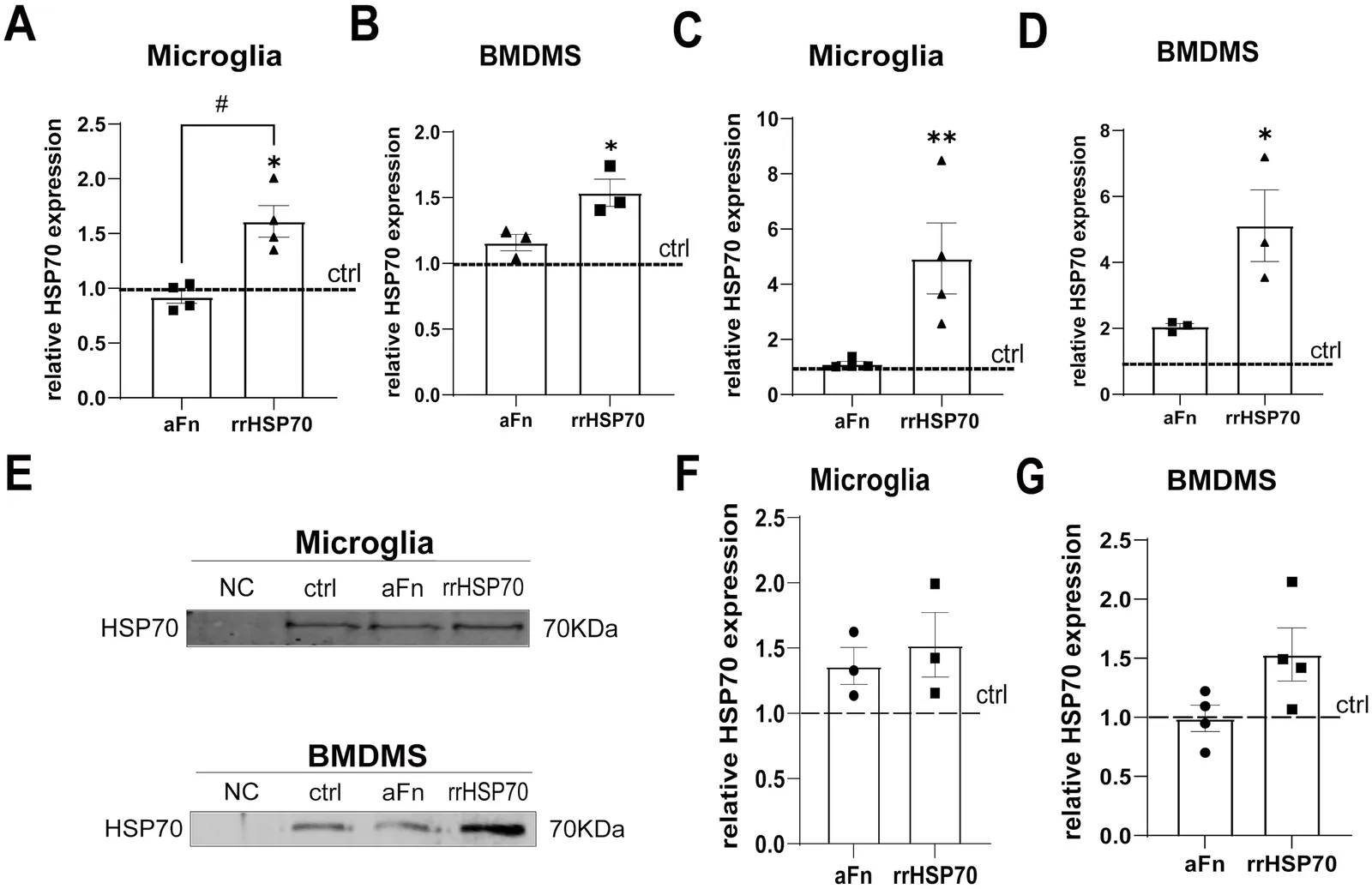
HSP70 expression in microglia/bone marrow-derived macrophages (BMDMs). Microglia/BMDMs were left unstimulated (ctrl), or incubated with rrHSP70 and fibronectin aggregates (aFn) respectively for 48 hours for TMT-labeled quantitative proteomic analysis. **(A, B)** The expression of HSP70 in microglia (A, n=4)/BMDMs (B, n=3) by TMT-labeled quantitative proteomic analysis. Each bar represents the mean ± SEM of three or four independent experiments. Statistical differences were assessed with the Kruskal-Wallis test (*p < 0.05). **(C, D)** The expression of HSP70 in Microglia (C, n=4)/BMDMs (D, n=3) by Parallel reaction monitoring (PRM) analysis. Each bar represents the mean ± SEM of four or three independent experiments. Statistical differences were assessed with the Kruskal-Wallis test (*p < 0.05). **(E)** Microglia/bone marrow-derived macrophages (BMDMs) were left unstimulated (ctrl), incubated with aFn or rrHSP70 for 24 hours and refreshed the media to culture for another 24 hours. The media were collected to assess HSP70 expression by western blot analysis. **(F, G)** The quantifications of HSP70 expression in microglia (F, n=3)/BMDMs (G, n=4) medium are shown. Each bar represents the mean ± SEM. Statistical differences were assessed with the Kruskal-Wallis test (*p < 0.05, ^#^p < 0.05).

In rrHSP70-incubated microglia, HSP70 (protein accession: P0DMW0) was upregulated as a secretable protein compared with aFn-incubated microglia, as shown by TMT-labeled quantitative proteomic analysis at a fold change >1.2 (**Supplementary Table 1**; **Figure 1A**). In contrast, aFn did not significantly regulate HSP70 (protein accession: P0DMW0) in microglia compared with the control at a fold change >1.2 (**Supplementary Table 1**; **Figure 1A**). In rrHSP70-incubated BMDMs, HSP70 (protein accession: P0DMW0) was upregulated compared with the control at a fold change >1.2 (**Supplementary Table 2**; **Figure 1B**). In aFn-incubated BMDMs, HSP70 (protein accession: P0DMW0) was not significantly regulated at a fold change >1.2. HSP70 was the most abundant secretable protein in rrHSP70-incubated microglia/BMDMs according to TMT-labeled quantitative proteomic analysis, indicating that rrHSP70, but not aFn, may regulate HSP70 expression in microglia/BMDMs.

To confirm the TMT-labeled quantitative proteomic results, PRM analysis was used to validate HSP70 expression in microglia/BMDMs. PRM analysis also showed that rrHSP70-incubated microglia/BMDMs expressed higher HSP70 levels than controls (**Figures 1C, D**). To determine whether microglia/macrophages secreted HSP70 extracellularly, serum-free culture media were collected for western blotting (**Figures 1E–G**). To remove residual rrHSP70 and aFn, microglia/BMDMs were incubated with rrHSP70 or aFn in serum-free culture media for 24 h; the media were then replaced and collected after another 24 h for western blotting. High extracellular HSP70 levels were detected in rrHSP70-incubated BMDM cultures compared with the control (**Figures 1E–G**), indicating that rrHSP70 may regulate HSP70 production and secretion.

### 3.2 rrHSP70 induces HSP70 expression in microglia/BMDMs, possibly through HSF-1 phosphorylation

To determine whether rrHSP70 activates signaling pathways that promote HSP70 expression in microglia/BMDMs, PTDM analysis was performed to assess protein phosphorylation. Because PTDM analysis required substantial protein and microglia/BMDMs showed similar protein-expression profiles, BMDMs were collected for PTDM analysis. In PTDM analysis, phosphorylation was upregulated in 427 proteins, whereas eight proteins showed reduced phosphorylation in rrHSP70-incubated BMDMs compared with control BMDMs (fold change >1.3). Because HSF-1 phosphorylation has been shown to activate HSP70 expression in glial cells (29, 30), PTDM analysis showed markedly increased HSF-1 phosphorylation in BMDMs after rrHSP70 incubation (**Table 2**).

**Table 2:** The differential phosphorylated protein of HSF-1 and phosphorylated proteins in Fc gamma R-mediated phagocytosis in bone marrow derived macrophage (BMDMS) by post-translational modification analysis.

Protein accession		Protein description	Gene name	Regulated type	Mean ± SEM (ctrl, n=2)	Mean ± SEM (rrHSP70, n=2)
F1MAF1		Heat shock transcription factor 1	HSF-1	Up	0.828±0.02	1.299±0.076
Fc gamma R-mediated phagocytosis	F1LMW7	Myristoylated alanine-rich C-kinase substrate	Marcks	Up	0.8165±0.111	1.187±0.118
	P04157	Receptor-type tyrosine-protein phosphatase C	Ptprc (pCD45)	Up	0.8265±0.063	1.078±0.057
	F7EWC1	Vasodilator-stimulated phosphoprotein	Vasp	Up	0.833±0.066	1.0865±0.067
	F1LSR2	GRB2-associated-binding protein 2	Gab2	Up	0.766±0.025	1.171±0.088
	A0A140UHX0	Protein kinase C delta type	Prkcd	Up	0.7785±0.06	1.056±0.045
	A0A0G2JXD7	Non-specific serine/threonine protein kinase	Akt3	Up	0.813±0.038	1.076±0.074
	P47196	RAC-alpha serine/threonine-protein kinase	Akt1	Up	0.813±0.083	1.076±0.074
	P47197	RAC-beta serine/threonine-protein kinase	Akt2	Up	0.813±0.038	1.076±0.074
	F1M981	Phosphatidylinositol-3,4,5-trisphosphate 5-phosphatase	Inpp5d	Up	0.8085±0.102	1.094±0.157
	Q6AYB2	RCG53912, isoform CRA_a	Sphk2	Up	0.792±0.076	1.1125±0.086
	P09216	Protein kinase C epsilon type	Prkce	Up	0.7465±0.017	1.108±0.044

**Figure 2:**
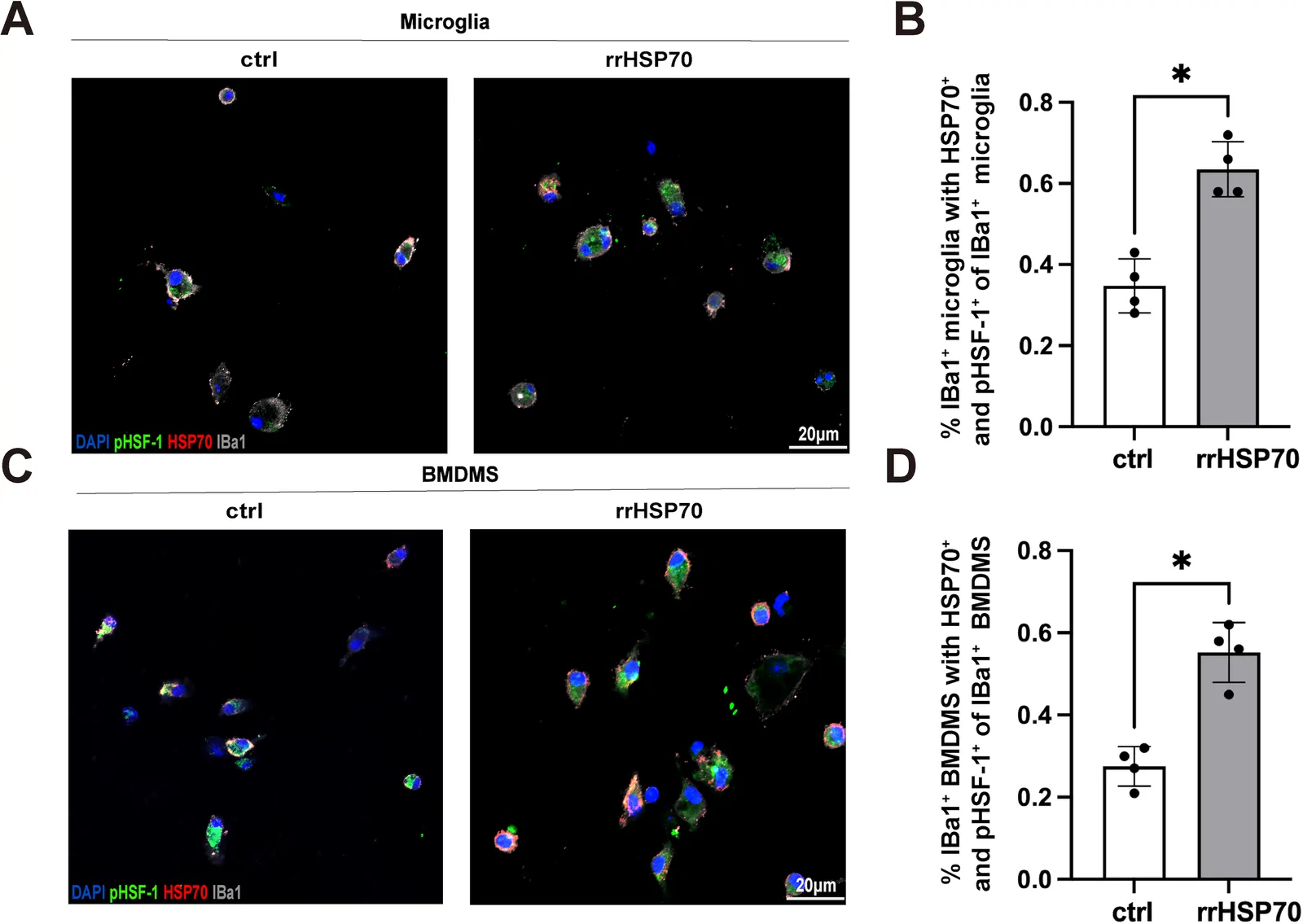
rrHSP70 active phosphorylation of HSF-1 to expression HSP70 in microglia/bone marrow-derived macrophages (BMDMs). Microglia/BMDMs were left unstimulated (ctrl), or incubated with rrHSP70 for 48 hours for immunofluorescent staining. **(A, C)**. Immunofluorescent staining of pHSF-1 (Green), HSP70 (Red) and IBa1 (Gray) in Microglia **(A)**/BMDMs**(C)**. Scale bars are 20 μm. **(B, D)**. The number of pHSF-1 and HSP70 within IBa1 positive cells are manually counted in four independent experiments. Statistical differences were assessed by the Mann-Whitney test (*p < 0.05, n=4).

To verify the PTDM results, immunohistochemistry was used to assess phosphorylated HSF-1 and HSP70 expression in rrHSP70-incubated microglia/BMDMs *in vitro* (**Figures 2A–D**). The analyses showed that phosphorylated HSF-1- and HSP70-positive cells increased in rrHSP70-incubated microglia/BMDMs compared with controls, consistent with the PTDM results (**Figures 2A–D**). These findings suggest that HSF-1 phosphorylation may contribute to HSP70 expression in microglia/BMDMs. Therefore, rrHSP70 may induce HSF-1 phosphorylation in microglia/BMDMs, thereby promoting HSP70 expression.

### 3.3 rrHSP70 induces phagocytosis in microglia/BMDMs through the Fc gamma R-mediated signaling pathway

PTDM analysis identified several major signaling pathways regulated in rrHSP70-incubated BMDMs, including insulin signaling, Fc gamma R-mediated phagocytosis, microRNAs in cancer, and apelin signaling pathways (**Supplementary Figure 1**; **Table 2**). These pathways were upregulated, suggesting that rrHSP70 may be phagocytosed by microglia/BMDMs, leading to HSF-1 phosphorylation and subsequent HSP70 expression.

**Figure 3:**
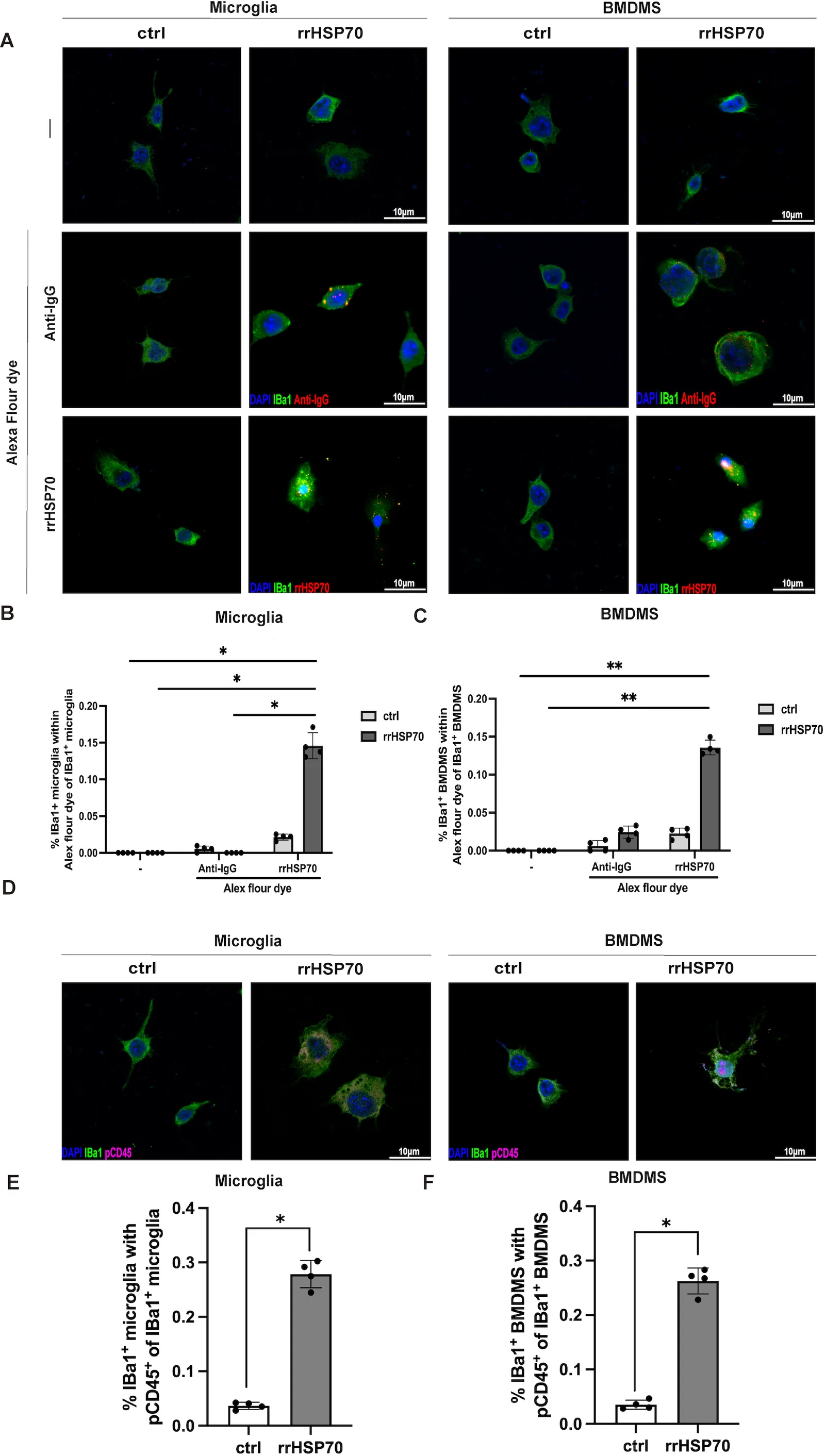
rrHSP70 induce phagocytosis of microglia/bone marrow-derived macrophages (BMDMs) and phosphorylation of CD45. **(A)** Immunofluorescent staining of IBa1 (Green), alexa 488-labeled rrHSP70 (Red) and alexa 488-conjugated goat anti-mouse (rat) IgG (Red). IBa1 positive cells with alexa 488-labeled rrHSP70 (Red) or the alexa 488-conjugated goat anti-mouse (rat) IgG (Red) indicate phagocytosis of cells. Scale bars are 10 μm. **(B, C)** The quantifications of IBa1 positive microglia with phagocytosis by immunofluorescent staining (n=4, in B). The number of IBa1 positive BMDMs with phagocytosis are manually counted in four independent experiments **(C)**. Each bar represents the mean ± SEM. Statistical differences were assessed with the Kruskal-Wallis test (*p < 0.05). **(D)** Immunofluorescent staining of IBa1 (Green) and pCD45 (Pink) in microglia/BMDMs. Scale bars are 10 μm. **(E, F)** The quantifications of IBa1 positive microglia with pCD45 by immunofluorescent staining (n=3, in E). The number of IBa1 positive BMDMs with pCD45 are manually counted in three independent experiments **(F)** Each bar represents the mean ± SEM. Statistical differences were assessed with the Mann-Whitney test (*p < 0.05, n=3).

To assess phagocytosis in rrHSP70-incubated microglia/BMDMs, dye-labeled rrHSP70 and dye-labeled anti-IgG were added after rrHSP70 incubation (**Figure 3**). Immunohistochemistry showed that both dye-labeled anti-IgG and rrHSP70 were phagocytosed by microglia/BMDMs after rrHSP70 incubation (**Figures 3A–C**). Furthermore, microglia/BMDMs showed markedly higher phagocytosis of dye-labeled rrHSP70 than controls, indicating that dye-labeled rrHSP70 may persistently stimulate phagocytosis in these cells.

CD45 (receptor-type tyrosine-protein phosphatase C, *Ptprc*) is a major receptor mediating phagocytosis in Fc gamma R-mediated signaling pathways, and its phosphorylation increased in PTDM analysis (**Table 2**). Therefore, CD45 phosphorylation in rrHSP70-incubated microglia/BMDM cultures was assessed by immunohistochemistry (**Figures 3D–F**). The analyses showed that phosphorylated CD45-positive cells increased in rrHSP70-incubated microglia/BMDMs compared with controls (**Figures 3D–F**). These findings suggest that rrHSP70 may induce microglia/BMDM phagocytosis through CD45 phosphorylation in Fc gamma R-mediated signaling pathways.

### 3.4 Blocking or inhibiting CD45 reduces microglial phagocytosis and HSF-1 phosphorylation

**Figure 4:**
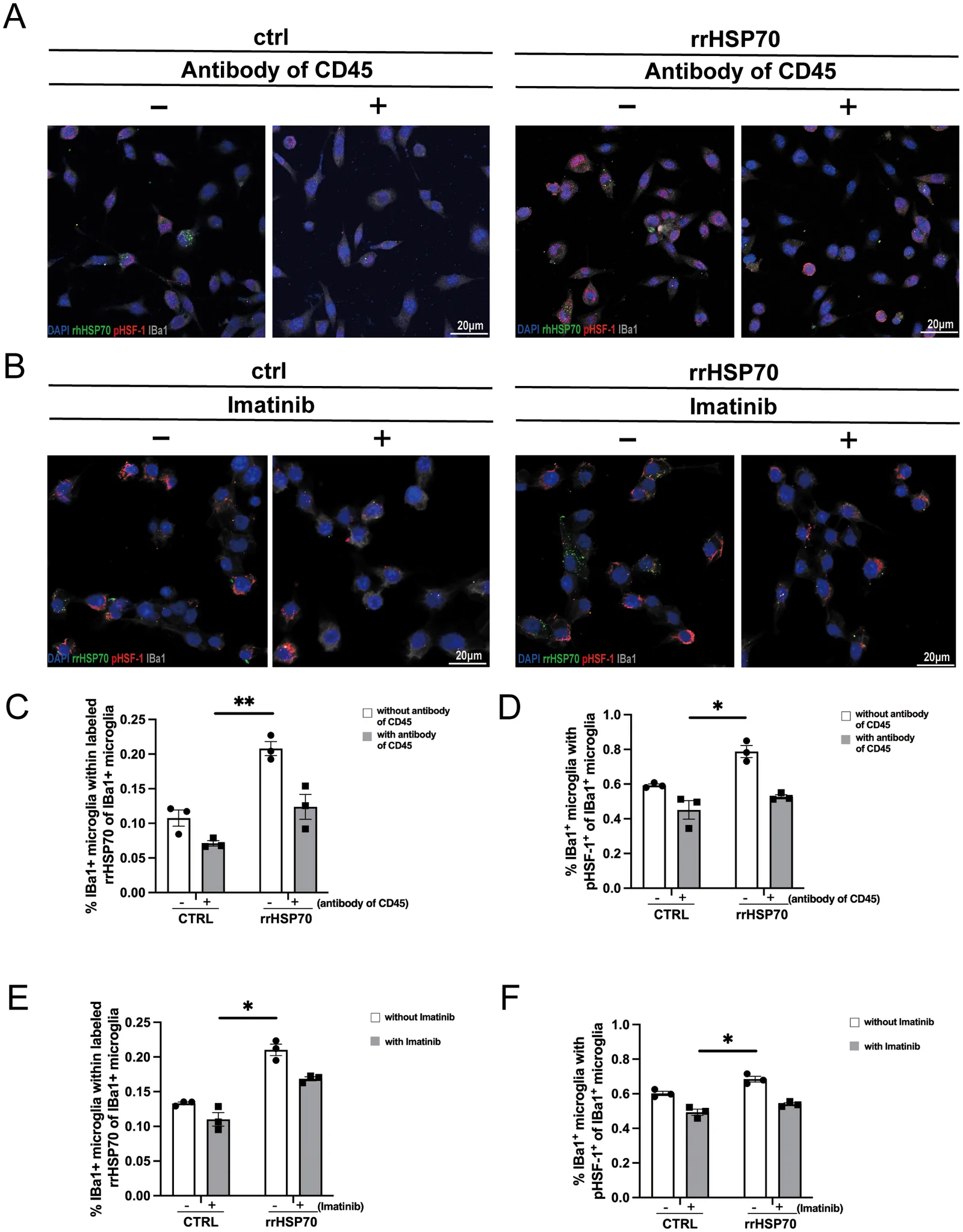
The reduction of the phagocytosis and the phosphorylation of HSF-1 in microglia by inhibiting the CD45. **(A)** Immunofluorescent staining of IBa1 (Gray), dye labeled rrHSP70 (Green) and pHSF-1 (Red) of blocking CD45 by antibody of CD45. IBa1 positive cells with alexa 488-labeled rrHSP70 (Red) indicate phagocytosis of cells. Scale bars are 20 μm. **(B)** Immunofluorescent staining of IBa1 (Green), dye labeled rrHSP70 (Green) and pHSF-1 (Red) of inhibiting CD45 by Imatinib. IBa1 positive cells with alexa 488-labeled rrHSP70 (Red) indicate phagocytosis of cells. Scale bars are 20 μm. **(C, E)** The quantifications of IBa1 positive microglia with phagocytosis by immunofluorescent staining are manually counted (n=3, in B). **(D, F)** The quantifications of IBa1 positive microglia with phosphorylation of HSF-1 (pHSF-1) by immunofluorescent staining are manually counted in three independent experiments **(C)**. Each bar represents the mean ± SEM. Statistical differences were assessed with the Kruskal-Wallis test (*p < 0.05).

To verify CD45 as a mediator of rrHSP70 phagocytosis in microglia, a CD45 antibody was used to block the receptor. Immunohistochemistry showed that CD45 blockade reduced dye-labeled rrHSP70 uptake in control and rrHSP70-incubated microglia compared with unblocked cells (**Figures 4A, B**). These results indicate that CD45 blockade inhibits dye-labeled rrHSP70 phagocytosis in microglia, suggesting that CD45 may mediate rrHSP70 uptake in these cells (**Figures 4A, B**). Moreover, CD45 blockade reduced phosphorylated HSF-1-positive microglia in both control and rrHSP70-incubated groups compared with unblocked cells (**Figures 4A–C**). Thus, CD45 blockade reduced both HSF-1 phosphorylation and rrHSP70 phagocytosis, suggesting that rrHSP70 may regulate phagocytosis through CD45, thereby modulating HSF-1 phosphorylation and HSP70 expression in microglia.

To determine whether CD45 activity functions upstream of Fc gamma R-mediated phagocytosis, Imatinib was used to inhibit CD45 activity. Immunohistochemistry showed that Imatinib reduced dye-labeled rrHSP70 uptake in rrHSP70-incubated microglia compared with Imatinib-treated controls (**Figures 4A, B**). These results indicate that Imatinib inhibited dye-labeled rrHSP70 phagocytosis in microglia, suggesting that CD45 activation may mediate rrHSP70 uptake in these cells (**Figures 4A, B**). Moreover, Imatinib reduced phosphorylated HSF-1-positive microglia in rrHSP70-incubated cells compared with Imatinib-treated controls (**Figures 4A, B**). These findings suggest that CD45 inhibition reduces both HSF-1 phosphorylation and rrHSP70 phagocytosis in microglia, indicating that rrHSP70 may regulate phagocytosis through CD45, thereby modulating HSF-1 phosphorylation and HSP70 expression in these cells.

### 3.5 rrHSP70 may regulate microglia/macrophages to promote OLG maturation

**Figure 5:**
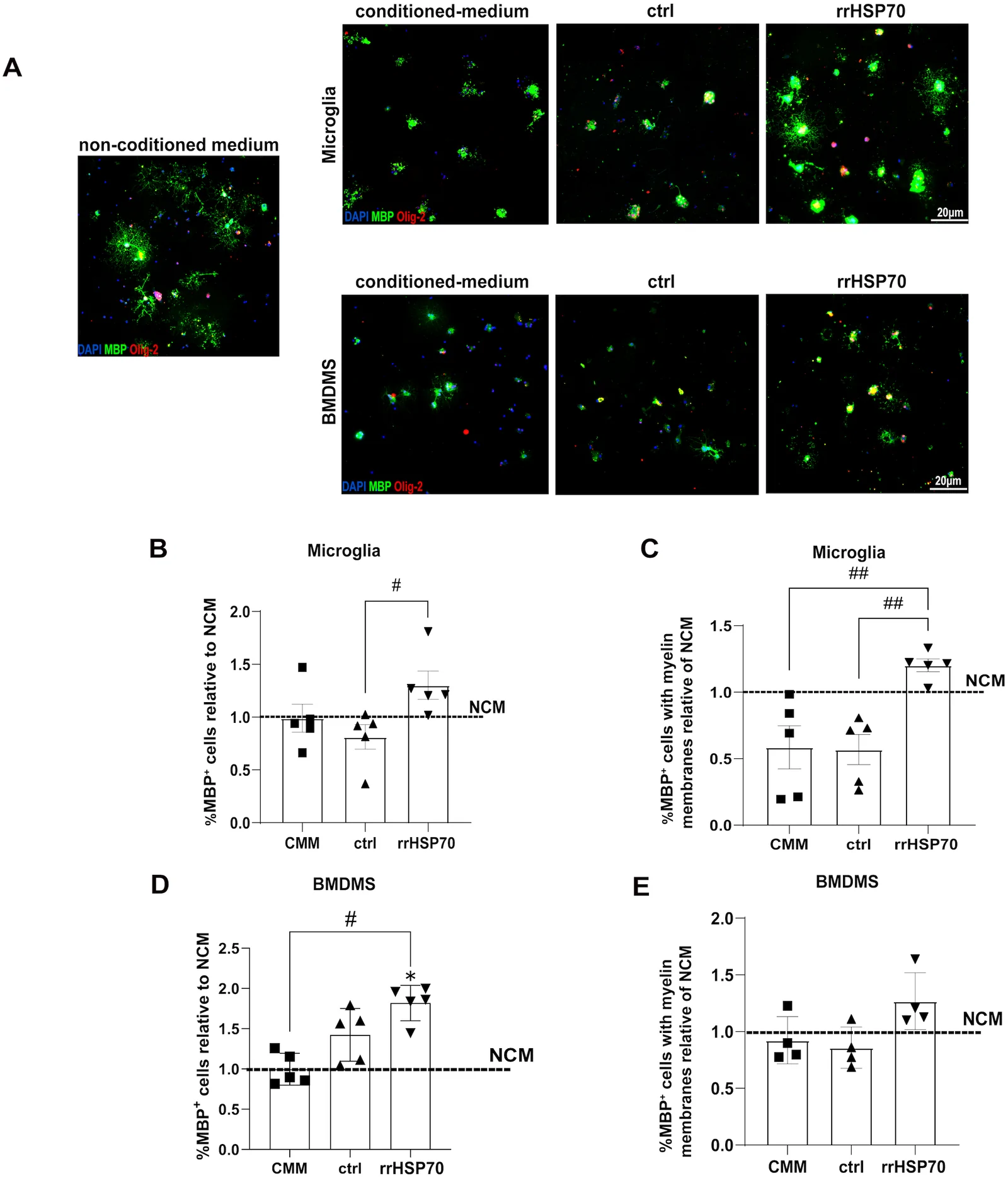
rrHSP70 incubated with microglia/bone marrow-derived macrophages (BMDMs) promote maturation of OLG. The microglia/BMDM-conditioned medium (CMM) was collected to culture oligodendrocyte progenitor cells (OPCs). Representative images are shown in **(A)**. Scale bar is 100 μm. The number of MBP-positive cells of total Olig2-positive cells was determined 3 days after initiating differentiation [Microglia, in **(B)**, n=5; BMDMs, in **(D)**, n=4]. MBP-positive cells bearing myelin membranes was determined 3 days after initiating differentiation [Microglia, in **(C)**, n=5; BMDMs, in **(E)**, n=4]. Bars represent mean expression levels versus non-conditioned medium which was set at 1 for each independent experiment (horizontal line). Error bars show the standard error of the mean (SEM). Statistical analyses were performed using the Kruskal-Wallis test (*p < 0.05).

As described above, microglia/macrophages produce HSP70, which may regulate OLG maturation. Therefore, we investigated whether exogenous rrHSP70 regulates OLG differentiation and maturation through microglia/BMDMs *in vitro*. Consistent with previous studies, OPCs cultured on PLL in unconditioned medium differentiated spontaneously (**Figure 5A**). In medium from rrHSP70-incubated microglia, MBP-positive cells (“differentiation”) significantly increased compared with control medium (ctrl) (**Figures 5A, B**). Furthermore, MBP-positive cells bearing myelin membranes (“maturation”) increased in medium from rrHSP70-incubated microglia compared with CMM and control medium (ctrl) (**Figures 5A, C**). These findings suggest that rrHSP70-incubated microglia may secrete factors that promote OLG maturation. In medium from rrHSP70-incubated BMDMs, MBP-positive cells (“differentiation”) increased compared with CMM and BMDM control medium (ctrl) (**Figures 5A, D**). Moreover, MBP-positive cells with myelin membranes (“maturation”) increased in medium from rrHSP70-incubated BMDMs compared with BMDM control medium (ctrl) (**Figures 5A, E**). Notably, OLGs cultured in medium from rrHSP70-incubated microglia showed more mature morphology than those cultured in medium from rrHSP70-incubated BMDMs. Therefore, HSP70 secreted by microglia/BMDMs may promote OLG maturation.

### 3.6 HSP70 expression increases during demyelination in a toxin-induced demyelination animal model

HSP70 expression was examined to determine whether it represents a natural response to demyelinating injury. HSP70 levels were assessed in cuprizone-induced demyelination animal models.

**Figure 6:**
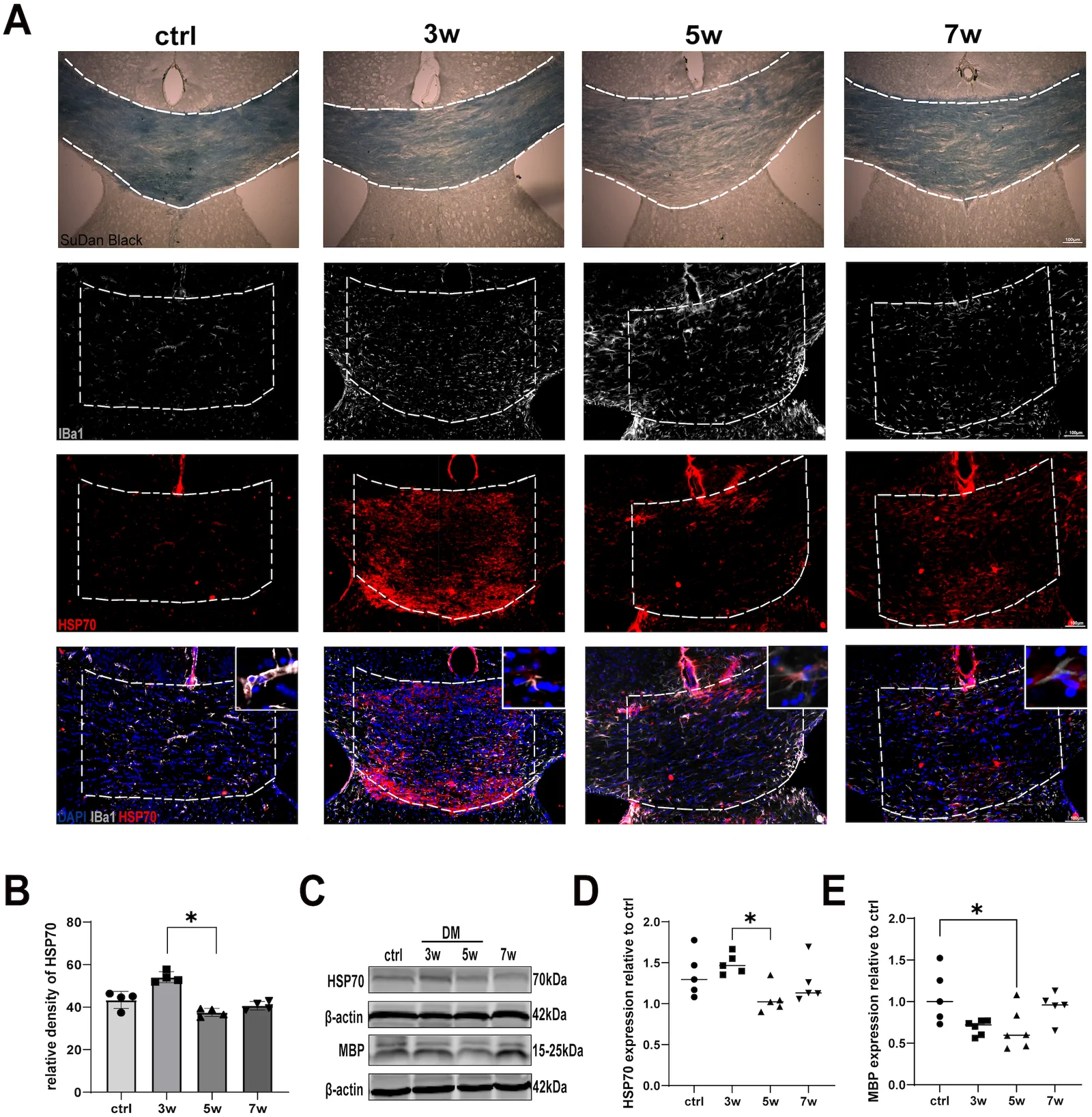
HSP70 expression in cuprizone-induced demyelination. **(A)** Sudan Black staining indicates the demyelination and remyelination in mice fed with 0.2% cuprizone demyelination model (n=4). Control mice (ctrl), mice fed with cuprizone for 3 or 5 weeks (3 or 5 w, demyelination), and mice fed with normal diets 2 weeks after 5 weeks cuprizone feeding (2 w, remyelination). Scale bar is 100 µm. Immunofluorescent staining of HSP70 (Red), IBa1 (Gray) in mice fed with 0.2% cuprizone demyelination model. Nuclei are indicated in blue. Scale bars are 100 μm. **(B)** The quantifications of HSP70 by immunofluorescent staining are shown in B (n=4). Each bar represents the mean ± SEM. Statistical differences were assessed with the Kruskal-Wallis test (*p < 0.05). Western blot showing MBP and HSP70 expression in total corpus callosum homogenates (100 µg) of mice indicated in **(C)** Actin serves as a loading control. Representative blots of five or six animals per condition are shown. D, and **(E)** Quantitative analysis of MBP **(E)**, and HSP70 **(D)** expression of **(G)** Bars depict mean ± SEM. Statistical analyses were performed using the Kruskal-Wallis test (*p < 0.05).

In the cuprizone model, demyelinating lesions were clearly observed in the corpus callosum at 3 and 5 weeks. Remyelination was observed 2 weeks after cuprizone withdrawal, consistent with previous studies (28), as reflected by the reappearance of Sudan Black staining (**Figure 6A**). Western blotting and immunohistochemical analyses showed increased HSP70 levels during early demyelination compared with late demyelination (**Figures 6A–E**). In particular, immunohistochemical analysis showed that HSP70 levels were markedly elevated only at 3 weeks (early demyelination phase) compared with 5 weeks (late demyelination phase) (**Figures 6A, B**). Western blotting also showed markedly increased HSP70 levels at 3 weeks compared with 5 weeks (**Figures 6C–E**). Western blotting and immunohistochemical analyses showed reduced HSP70 levels at 5 weeks compared with the control, 3-week time point, and 7-week time point (remyelination phase) (**Figures 6A–E**). During remyelination at 7 weeks, HSP70 levels returned to control-like levels (**Figures 6A, C**). Moreover, IBA1-positive microglia/macrophages were the main cellular source of HSP70 expression (**Figure 6A**). Therefore, the role of HSP70 in remyelination may be underestimated. HSP70 was highly expressed in early demyelinated lesions, potentially promoting microglia/macrophage phagocytosis to clear factors that inhibit remyelination. HSP70 levels decreased during late demyelination and returned to control-like levels during remyelination, suggesting a potential role for microglia/macrophages in regulating OPC proliferation and recruitment and promoting OLG maturation during remyelination. Therefore, the cuprizone-induced demyelination model is suitable for exogenous rrHSP70 administration to examine the role of HSP70 in remyelination.

### 3.7 rrHSP70 may promote OLG maturation and remyelination in toxin-induced demyelination models

**Figure 7:**
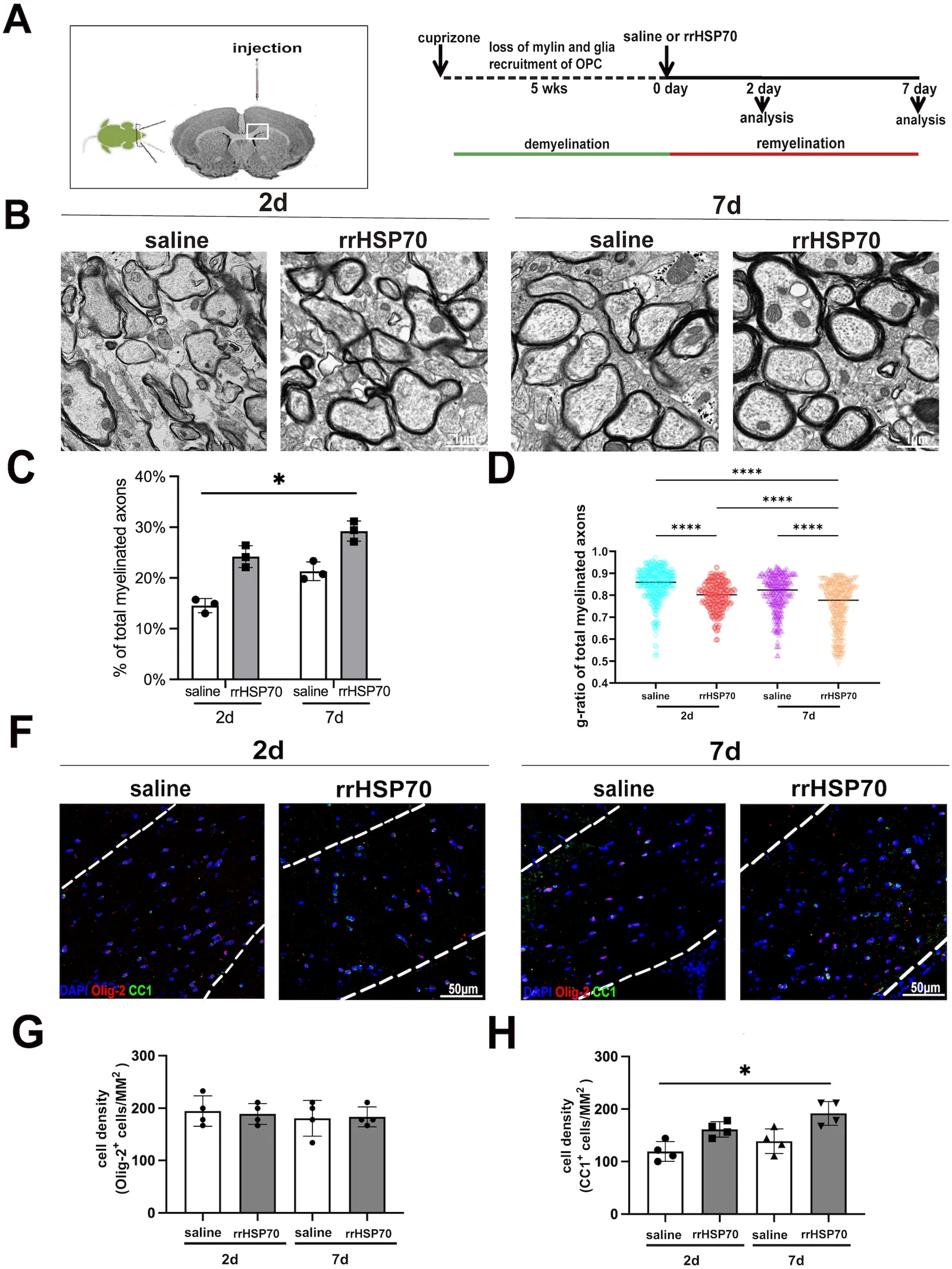
Exogenous addition of rrHSP70 promotes remyelination and maturation of OLG in cuprizone-induced demyelination animal model. **(A)** Schematic representation of injection rrHSP70 in initiation of remyelination in cuprizone-induced demyelination mice model. **(B)** Images are representative electromicrographs of remyelinated lesion center on saline (left) and rrHSP70 injection (n=3). **(C)** Myelinated axons as a percentage of total axons in each field of view. Each bar represents the mean ± SEM. Statistical differences were assessed with the Kruskal-Wallis test (*p < 0.05). **(D)** The g-ratio of individual axons were plotted against the corresponding axon diameters. Scale bar in B = 1 μm. Statistical differences were assessed with a one-way analysis of variance (ANOVA), followed by the Tukey’s multiple comparisons test (*p < 0.05, **p < 0.01, ***p<0.001). **(F)** Immunofluorescent staining of olig-2 (Red), and CC1 (Green) in remyelination in mice fed with 0.2% cuprizone demyelination model. Nuclei are indicated in blue. Scale bars are 20 μm. **(G, H)** The quantifications of olig-2 positive cells **(G)**, and CC1 positive cells **(H)** by immunofluorescent staining (n=4). The number of olig-2 and CC1 positive cells are manually counted in four animal lesions. Each bar represents the mean ± SEM. Statistical differences were assessed with the Kruskal-Wallis test (*p < 0.05).

In the cuprizone-induced demyelination animal model, saline or rrHSP70 was injected intralesionally into the corpus callosum at remyelination onset (day 0) (**Figure 7A**). Because remyelination robustly recovered after toxin-induced demyelination, the effect of a single rrHSP70 injection at remyelination onset was assessed at 2 and 7 days post-lesion (DPL). Electron microscopy analysis at 2 and 7 DPL showed that remyelination progressed from 2 to 7 DPL in rrHSP70-injected lesions (**Figure 7B**), as reflected by a significantly increased percentage of myelinated axons and significantly decreased g-ratios (**Figure 7C**). These results suggest that rrHSP70 may enhance early remyelination in toxin-induced demyelination models. Because OLGs support remyelination, their maturation was also assessed in this animal model. Immunohistochemistry at 2 and 7 DPL after injection showed that Olig2-positive cell numbers, representing OLG lineage cells, were similar between rrHSP70-injected lesions and saline-injected controls (**Figures 7F, G**). To assess whether Olig2-positive cells differentiated into mature OLGs, CC1-positive cells, a marker of mature OLGs, were analyzed at 2 and 7 DPL. At 7 DPL, rrHSP70-injected lesions showed more mature OLGs, defined as Olig2- and CC1-positive cells, than saline-injected control lesions (**Figures 7F–H**). Olig2-positive cell numbers in rrHSP70-injected lesions remained similar from 2 to 7 DPL (**Figures 7F, G**), whereas CC1-positive cells significantly increased from 2 to 7 DPL (**Figures 7F–H**), suggesting that rrHSP70 may promote OLG maturation. These data suggest that rrHSP70 may stimulate OLG maturation and facilitate remyelination in the cuprizone-demyelinated corpus callosum. Therefore, rrHSP70’s role in OLG development was assessed in subsequent experiments.

### 3.8 rrHSP70 may promote OLG maturation by enhancing MBP expression and facilitating phosphorylated MBP redistribution

**Figure 8:**
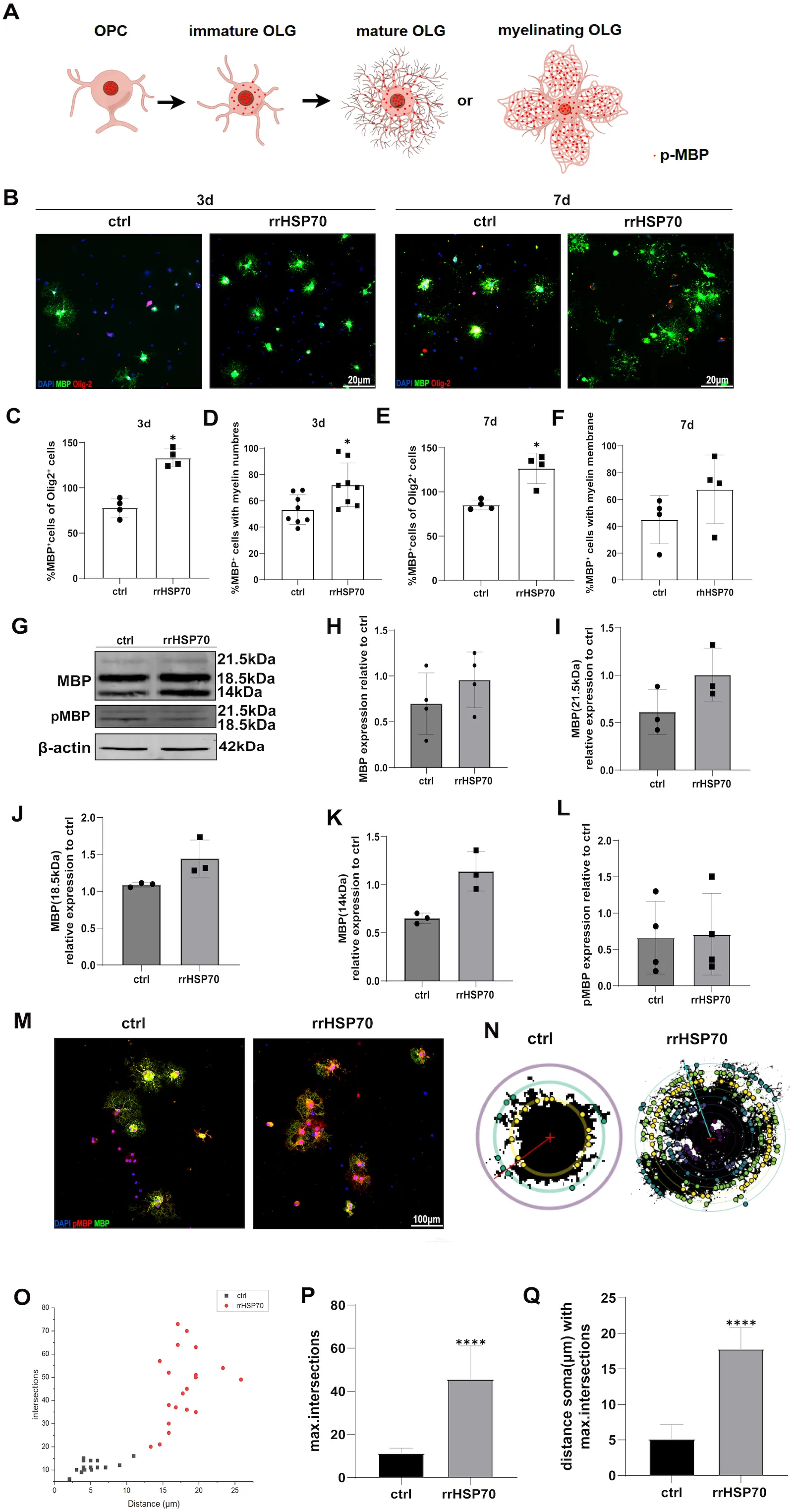
Exogenous addition of rrHSP70 promotes maturation of OLG *in vitro*. **(A)** Schematic representation of OPCs differentiation and maturation *in vitro*. **(B)** Representative images of MBP and Oligo-2 immunocytochemistry of OLGs cultured on PLL at 3 days or 7 days. Scale bar is 100 μm. The number of MBP-positive cells [differentiation, 3 days in **(C)**, and 7 days in **(E)**], the number of MBP-positive cells bearing myelin membranes [‘myelination’, 3 days in **(D)**, and 7 days in **(F)**] were assessed. Each bar represents the mean ± SEM of four or eight independent experiments. Statistical differences were assessed with the Mann-Whitney test (* p < 0.05, n≥4). **(G)** OLGs were incubated with rrHSP70 for 3 days to assess expression of MBP and pMBP by western blot analysis. **(H–L)** The quantifications of expression of MBP and pMBP in OLGs are shown. Each bar represents the mean ± SEM. Statistical differences were assessed with the Mann-Whitney test (* p < 0.05, n=3). **(M)** Representative images of MBP and pMBP immunocytochemistry of OLGs. Scale bar is 100 μm. **(N)** The representative image of the sholl analysis. **(O)** The number of processes that intersect with the concentric circles of the sholl analysis as a function of the distance from the soma. P, and **(Q)** Maximum intersections **(P)**, distance of the soma with the maximum number of branch points **(Q)**. Each bar represents the mean ± SEM of four independent experiments. Statistical differences were assessed with the Mann-Whitney test (*p < 0.05, n=4).

To clarify whether rrHSP70 directly stimulates OLG differentiation and maturation *in vitro*, its effect on OLG differentiation and maturation was examined (**Figure 8A**). OLG monocultures were grown on PLL with or without different rrHSP70 concentrations (**Supplementary Figure 2**; **Figure 8B**). OLG differentiation and maturation were assessed based on the percentage of MBP-positive cells and the proportion of cells with elaborate myelin membranes, respectively. Among the tested rrHSP70 concentrations, 500 ng/mL was most effective in promoting OLG maturation (**Supplementary Figures 2A–C**). Therefore, morphologically immature OLGs were incubated with 500 ng/mL rrHSP70 or without rrHSP70 for 3 or 7 days. Furthermore, MBP-positive OLGs significantly increased in rrHSP70-incubated cells compared with controls after 3 days (**Figure 8B**). MBP-positive cells forming myelin membranes were also increased in rrHSP70-incubated cells compared with controls, indicating that rrHSP70 may promote OLG differentiation and maturation (**Figures 8B–F**). After 7 days, MBP-positive OLGs increased in rrHSP70-incubated cells compared with controls (**Figures 8B, E, F**). OLGs incubated with rrHSP70 were morphologically mature, and MBP-positive OLGs forming myelin membranes were markedly increased in rrHSP70-incubated cells compared with controls after 7 days (**Figures 8B, F**). These findings indicate that sustained rrHSP70 exposure likely promoted OLG differentiation and maturation, consistent with the animal-model results.

To determine which molecules rrHSP70 regulated to promote OLG maturation, MBP expression and MBP phosphorylation (pMBP) were assessed in OLGs after 3 days of rrHSP70 treatment (**Figures 8G–L**). Western blotting showed increased MBP expression in rrHSP70-incubated OLGs compared with controls (**Figure 8G**). In particular, 18.5-kDa MBP and 14-kDa MBP expression increased in rrHSP70-incubated OLGs compared with controls (**Figures 8G, J, K**). Myelinogenesis phases are closely associated with pMBP, which stabilizes myelin sheath structure (31–34). However, pMBP levels did not differ significantly between rrHSP70-incubated OLGs and controls (**Figure 8L**). To assess whether rrHSP70 redistributes pMBP in differentiated OLGs, pMBP distribution within cellular processes was traced in differentiated OLGs under control and rrHSP70-incubated conditions. Sholl analysis was then performed to quantify pMBP-positive process intersections relative to radial distance from the soma center (**Figures 8M–Q**). Sholl analysis showed a higher maximum number of pMBP-positive process intersections in differentiated OLGs under rrHSP70-incubated conditions compared with controls (**Figures 8N–Q**). Similarly, differentiated OLGs treated with rrHSP70 showed a greater radial distance of pMBP-positive process intersections from the soma center than controls. This indicates that rrHSP70-incubated differentiated OLGs were more mature than controls and that pMBP was distributed more extensively in the plasma membrane than in the soma center (**Figures 8N–Q**). These results indicate that although phosphorylated MBP levels did not differ significantly between rrHSP70-incubated OLGs and controls, pMBP distribution shifted from the cytoplasm to the plasma membrane in rrHSP70-incubated differentiated OLGs compared with controls. Overall, these results suggest that rrHSP70 may regulate MBP expression and pMBP redistribution to promote OLG maturation.

## 4 Discussion

“HSP70” refers to the endogenously expressed protein, whereas “rrHSP70” denotes the exogenously supplied recombinant protein. In this study, rrHSP70 was phagocytosed by microglia/BMDMs, leading to HSF-1 phosphorylation, potentially via the Fc gamma R-mediated signaling pathway, thereby maintaining HSP70 expression *in vitro*. HSP70 levels increased during early demyelination and returned to control-like levels in a toxin-induced demyelination animal model, indicating that HSP70 may regulate demyelination and remyelination. Microglia/macrophages were the main cellular source of HSP70 in toxin-induced demyelination animal models. rrHSP70 may promote OLG differentiation and maturation in primary cell cultures and toxin-induced demyelination animal models. Thus, because HSP70 may act context dependently to promote remyelination in toxin-induced demyelination models, local HSP70 modulation could be a promising strategy for enhancing remyelination in MS.

Because HSP70 is a highly conserved molecule, recombinant human HSP70 (rhHSP70) stimulates rat microglial/macrophage phagocytosis of amyloid-β (35). Murine HSP70 also enhances mouse macrophage phagocytosis (36, 37), indicating that rhHSP70 and murine HSP70 can regulate microglial/macrophage phagocytosis across species. Increased CD68 and CD163 expression reflects phagocytosis and pro-inflammatory factor production, respectively (36). In this study, microglia/BMDMs phagocytosed rrHSP70. CD45 was phosphorylated, and Fc gamma R-mediated phagocytosis signaling pathways were activated by rrHSP70. CD45 inhibition reduced rrHSP70-induced phagocytosis and HSF-1 phosphorylation in microglia, indicating that CD45 is likely a major receptor in Fc gamma R-mediated rrHSP70 phagocytosis in microglia. HSF-1 phosphorylation is a key regulator of HSP70 expression (29, 30). In this study, HSP70 expression was associated with HSF-1 phosphorylation. Notably, in rrHSP70-phagocytosing microglia/BMDMs, both HSF-1 phosphorylation and HSP70 expression increased. CD45 inhibition in microglia reduced HSF-1 phosphorylation and HSP70 expression, indicating that rrHSP70 phagocytosis by microglia/BMDMs may phosphorylate HSF-1 to drive HSP70 expression. Several studies have shown that stress conditions trigger HSP70 release from glial cells and peripheral blood mononuclear cells (38–40). However, our proteomic analysis showed that exogenous rrHSP70 may regulate HSP70 production and release by microglia/BMDMs under normal cell-culture conditions. Released HSP70 can trigger Toll-like receptor (TLR-2 and TLR-4) and CD14 co-stimulatory signaling during immune responses (41).

HSP70 is considered an attractive therapeutic target for MS because it participates in immune responses and neuroprotection. Previous studies have demonstrated that HSP70 is primarily produced by glial cells in the CNS (21, 22). Our results confirmed that microglia/macrophages produce HSP70 in a toxin-induced demyelination model. In this study, immunohistochemical analysis and western blotting showed elevated microglia/macrophage-derived HSP70 expression during early demyelination in a toxin-induced model. However, HSP70 expression returned to control-like levels during remyelination, indicating that its upregulation may reflect inflammation and immune activation during demyelination. During demyelination, MBP is released from myelin sheaths (42), and HSP70 expression is upregulated in inflammatory MS lesions (43, 44). In inflammatory demyelination, particularly in MS, HSP70 tends to form complexes with MBP, enhancing MBP phagocytosis by immune cells (19). As resident CNS immune cells, microglia are the primary cells that phagocytose HSP70–MBP complexes during demyelination. HSP70 chaperones proteins into the cytosol to facilitate antigen processing and presentation via major histocompatibility complex molecules (45). Human glial cells, especially OLGs, treated with pro-inflammatory cytokines stimulate HSP70 expression (46). This suggests that the inflammatory phase during early MS/EAE demyelination creates a preconditioning microenvironment that enables glial cells to produce and release HSP70, thereby supporting neuronal protection during demyelination. Consistently, our study showed that HSP70 expression increased during early demyelination and returned to control-like levels during remyelination. In the cuprizone-induced demyelination animal model, most mature OLGs undergo apoptotic death as early as 3 weeks of demyelination (47, 48). Meanwhile, myelin loss begins at weeks 2–3 and peaks around week 5 of cuprizone feeding. Numerous bipolar OPCs, representing a more undifferentiated morphology, are present during weeks 1–3 of cuprizone administration. By week 3, OPCs differentiate into star-like precursors, reflecting a more differentiated morphology, and peak during weeks 4–5 of cuprizone administration. During this process, microglia/macrophages accumulate alongside increasing OPC numbers and secrete cytokines and growth factors that support OPC recruitment and proliferation in the cuprizone-induced demyelination animal model. Microglia/macrophages create a remyelination-favorable microenvironment by phagocytosing debris during early demyelination and supporting OPC recruitment and proliferation during late demyelination. Therefore, transient HSP70 overexpression by microglia/macrophages at 3 weeks may chaperone myelin proteins, promote their immunogenic phagocytosis by microglia/macrophages, and protect neurons in toxin-induced demyelination animal models.

HSP70 expression and release are associated with the demyelination–remyelination process, suggesting distinct roles for HSP70 during demyelination and remyelination. HSP70 can also act as an anti-inflammatory factor by suppressing pro-inflammatory responses in inflammatory diseases (49). Thus, heat shock proteins, including HSP70, may exert immunostimulatory or immunosuppressive effects depending on context (50, 51). Because microglia/macrophage phenotypic transitions can support remyelination during the repair phase, HSP70 may contribute to OLG development during remyelination rather than primarily enhancing microglia/macrophage phagocytosis. This interpretation is consistent with reduced HSP70 levels at 5 weeks (late demyelination phase) and during remyelination in the demyelination animal model.

Studies have also shown that HSP70 is a crucial regulator of adult OLG differentiation and remyelination (26). rhHSP70 reduces biochemical storage levels, improves motor function, and extends life span in several neurological mouse models of LSDs (52). rhHSP70 improves myelination and OLG maturation in the cerebellum of a genetically modified mouse model (25). In this study, a single rrHSP70 injection at remyelination onset suggested that rrHSP70 likely promoted OLG maturation. At later stages after injection, rrHSP70 may further enhance OLG maturation, consistent with the primary cell culture results. Studies have suggested that OLG differentiation correlates with the nuclear localization of HSP70 (26). Our *in vitro* results revealed that 3-day rrHSP70 incubation may promote OLG development. Moreover, 7-day rrHSP70 incubation markedly promoted OLG differentiation and maturation. Because HSP70 is a highly conserved molecule, rhHSP70 and rrHSP70 may both regulate OLG maturation and remyelination. Overall, HSP70 overexpression during demyelination contributes to microglia/macrophage phagocytosis of myelin debris, immune responses, and neuronal protection. In contrast, rrHSP70 may contribute to remyelination by promoting OLG development, thereby further enhancing remyelination.

MBP contributes to myelin sheath stability and integrity through interactions with membranes and other proteins. MBP can link actin filaments and microtubules to the OLG membrane and regulate signal transduction in OLGs and myelin. MBP has four isoforms, 21.5-, 18.5-, 17-, and 14-kDa, in rodents and humans (32, 33). In this study, MBP expression in OLGs was enhanced by rrHSP70, indicating that rrHSP70 may regulate MBP expression by promoting OLG maturation. Myelinogenesis phases are closely associated with pMBP, which stabilizes myelin sheath structure, prevents proteolysis, and alters its interaction with phospholipid bilayers (31–34). During OLG maturation, pMBP redistributes to distinct ‘‘membrane-ruffled’’ regions of the plasma membrane, where it co-localizes with actin and tubulin. In this study, rrHSP70 did not substantially alter pMBP levels but redistributed pMBP from the cytoplasm to distinct ‘‘membrane-ruffled’’ regions. HSP70 can bind MBP and PLP, which may be associated with MBP and pMBP redistribution. Based on these observations, exogenous HSP70 administration into the CNS may be a potential therapeutic strategy for rescuing remyelination in MS. This is not only because HSP70 overexpression inhibits inflammatory factor production by astrocytes and microglia (53, 54) but also because exogenous HSP70 modulates microglia/macrophages in ways that may promote OLG maturation and remyelination.

In this study, animal models and several primary cell cultures were used to show that rrHSP70 is involved in regulating demyelination and remyelination. rrHSP70 may promote remyelination and OLG maturation. In primary OLG cultures, rrHSP70 appeared to promote MBP expression and pMBP redistribution during OLG development, which may explain how it regulates OLG maturation to support remyelination in toxin-induced demyelination animal models. Moreover, the results showed that rrHSP70 may enhance HSP70 expression in microglia/macrophages through HSF-1 phosphorylation. TMT-labeled quantitative proteomic and PTDM analyses showed that rrHSP70 appeared to activate the Fc gamma R-mediated signaling pathway in microglia/macrophages to facilitate its own phagocytosis, leading to HSF-1 phosphorylation and subsequent HSP70 expression. After microglia/macrophages were incubated with rrHSP70, rrHSP70 was phagocytosed *in vitro*, and CD45 was phosphorylated to mediate the Fc gamma R-mediated signaling pathway. Although rrHSP70 may promote OLG maturation to support remyelination, the precise mechanism by which rrHSP70 promotes OLG maturation and functional validation of remyelination, such as behavioral motor assessments or electrophysiological analyses, remain to be investigated. In addition, immature OLGs, such as PDGFRA^+^ OPCs, were not quantified in this study. Moreover, the sample sizes in each experimental group may limit the statistical robustness of the analyses, although this limitation was consistent with animal welfare principles. Although evidence shows that rrHSP70 induces microglia/macrophage phagocytosis and promotes remyelination, these findings require confirmation using genetically modified animal models.

## Data Availability

The datasets presented in this study can be found in online repositories. The names of the repository/repositories and accession number(s) can be found in the article/**Supplementary Material**.

## Supplementary material

Supplementary Figure 1 Signaling pathways regulated by rrHSP70 were predicted in Bone marrow-derived macrophages (BMDMs) by post-translational modification analysis.

Supplementary Figure 2 The influence of exogenous addition of different concentration of rrHSP70 on the differentiation and maturation of OPCs *in vitro*. **(A)** Representative images of MBP of OLGs cultured on PLL at 3 days after incubation are shown in **(A)**. Scale bar is 100 μm. **(B, C)** The number of MBP-positive cells [differentiation, 3 days in **(B)**], the number of MBP-positive cells bearing myelin membranes [‘myelination’, 3 days in **(C)**] were assessed at 3 days after incubation with rrHSP70. Each bar represents the mean ± SEM of four independent experiments. Statistical differences were assessed with the Kruskal-Wallis test (*p < 0.05).
